# Latent and active tuberculosis development in patients with rheumatoid arthritis receiving biologic disease-modifying antirheumatic drugs: A single-center prospective study

**DOI:** 10.1371/journal.pone.0295048

**Published:** 2024-01-11

**Authors:** Binh Bui Hai, Tuan Le Anh, Phuong Nguyen Thi Thu, Hung Nguyen Van, Giap Vu Van, Dung Hoang Van

**Affiliations:** 1 Centre of Rheumatology, Bach Mai Hospital, Hanoi, Vietnam; 2 Department of Rheumatology and Endocrinology, Thanh Hoa Hospital, Thanh Hoa, Vietnam; 3 Pharmacy Faculty, Hai Phong University of Medicine and Pharmacy, Hai Phong, Vietnam; 4 Department of Pharmacy, Hai Phong International Hospital, Hai Phong, Vietnam; 5 Department of Internal Medicine, Hanoi Medical University, Hanoi, Vietnam; 6 Department of Internal Medicine, Hai Phong International Hospital, Hai Phong, Vietnam; Osaka Metropolitan University, JAPAN

## Abstract

Biologics have revolutionized the treatment of rheumatoid arthritis (RA) in recent years. However, data from clinical trials and actual clinical practice have shown that biologics currently in use may constitute a risk factor for reactivation of tuberculosis (TB) in patients with latent TB infection. Therefore, screening for latent and active TB infection is mandatory before initiating biologic therapy in patients with RA. This prospective study aimed to analyze the clinical characteristics of patients with RA receiving biologic disease-modifying antirheumatic drugs at Bach Mai Hospital, Vietnam, between 2017 and 2022, and to identify factors affecting the occurrence of active and latent TB infection among these patients. Over a 12-month follow-up period, latent TB infection was confirmed in 20% of the total 180 included patients, while 3 (1.7%) patients developed active TB (one case of pulmonary, pleural, and gluteal TB each). History of TB risk factor exposure and lack of education were significantly associated with the occurrence of active and latent TB infection, with odds ratios (95% confidence intervals [CIs]) of 1.98 (1.78; 2.2) and 1.45 (1.31; 1.6), respectively. Follow-up duration and number of X-ray, computed tomography, bronchoscopy, and sputum acid-fast bacteria examinations were identified as factors that can aid in the early diagnosis of latent TB, with odds ratios (95% CIs) of 1.00 (1; 1.01), 1.02 (1; 1.05), 1.12 (1.11; 1.2), 1.11 (1.09; 1.2), and 1.13 (1.09; 1.17), respectively. Our study showed that, in countries with high TB burden like Vietnam, latent TB infection has high prevalence among patients with RA. We also provide useful information for the screening, monitoring, and treatment of latent and active TB infection in patients with RA.

## Introduction

Rheumatoid arthritis (RA) is a ubiquitous long-term autoimmune disorder, with a prevalence of approximately 1% globally, 0.5–1% in European countries, and 0.17–0.3% in Asian countries [1–3]. RA causes joint cartilage and bone damage resulting in ankylosis, which leads to disability with attendant decline in physical function and quality of life as well as increased risk of cumulative comorbid conditions. Two sets of diagnostic criteria are widely used in clinical practice to support clinician and patient decision-making, including the 1987 American College of Rheumatology (ACR) classification criteria and the 2010 ACR/European League Against Rheumatism (EULAR) classification criteria, recently updated by the ACR in 2021 [4–7].

In recent years, RA treatment is mainly based on disease-modifying antirheumatic drugs (DMARDs) due to their effectiveness, availability, and low cost [8,9]. DMARDs are classified into three groups, as follows: 1) conventional synthetic DMARDs (hydroxychloroquine, sulfasalazine, methotrexate, and leflunomide); 2) biologic DMARDs, including tumor necrosis factor (TNF) inhibitors (etanercept, adalimumab, infliximab, golimumab, and certolizumab pegol), T-cell costimulatory inhibitor (abatacept), interleukin (IL)-6 receptor inhibitors (tocilizumab and sarilumab), and anti-CD20 antibody (rituximab); and 3) targeted synthetic DMARDs, including Janus kinase inhibitors (tofacitinib, baricitinib, and upadacitinib) [10]. Several developments during the past two decades have changed the treatment of RA. These include the emergence of methotrexate as a first-line drug in the treatment of early RA, the development of new highly effective biologic products that can be used as monotherapy or in combination with methotrexate, and the demonstrated superiority of a combination DMARD regimen over methotrexate alone [11].

Although biologics have revolutionized the treatment of RA in the past decade, data from clinical trials and actual clinical practice have shown that biologics currently in use may constitute a risk factor for reactivation of tuberculosis (TB) in patients with latent TB infection [12]. Latent TB infection has been diagnosed in 10% of patients with RA when starting therapy with biologic DMARDs [13]. Therefore, screening for latent and active TB infection is mandatory before initiating biologic therapy in these patients [14,15]. In Vietnam, biologic DMARDs have been used since 2010; however, to date, no study has analyzed the overall risk of developing active and latent TB infection in patients with RA.

Therefore, this prospective study aimed to 1) characterize active and latent TB infection in patients with RA receiving biologic DMARDs alone, and 2) identify factors affecting the occurrence of active and latent TB infection in these patients.

## Materials and methods

### Study design and population

This was a prospective observational study with a follow-up period of 12 months after the initiation of biologic DMARD therapy. The study was conducted at the Center of Rheumatology of Bach Mai Hospital, Vietnam from December 1, 2017 to December 30, 2022. We included all patients who were diagnosed with RA according to the 1987 ACR and/or 2010 ACR/EULAR criteria with an indication for treatment with a TNF-α antagonist or an IL-6 inhibitor. Patients who refused to participate in the study and those who did not comply with the treatment were excluded.

### Data collection and study procedures

For all patients, the following data were collected: 1) demographics (age, sex, weight, height, and body mass index); 2) clinical characteristics (time from RA diagnosis, duration of morning stiffness, joint pain intensity, disease activity status, comorbidities); 3) RA treatment-related data (DMARD regimen before starting biologics, duration of conventional DMARD therapy, dosage of corticosteroids before starting biologics, and introduced biologics); and 4) TB screening-related data (related risk factors, laboratory tests, imaging results, follow-up duration, time to rescreening, and treatment regimens).

Joint pain intensity was assessed using the visual analogue scale (VAS). Patients were asked to indicate their pain intensity according to their subjective feeling at the time of evaluation on a 10-cm line divided with marks into 10 sections, each spaced 10 mm [16]. Based on the VAS scores, pain intensity was classified into three levels: mild (10–40 mm), moderate (40–60 mm), or severe (60–100 mm).

Disease activity status was assessed using the disease activity score-28 for RA (DAS-28). We employed the recently developed alternative calculation of the DAS-28 based on C-reactive protein (CRP) levels [17,18] using the following formula:

$$DAS28(CRP)=0.56\times \sqrt{\left(TJC28\right)}+0.28\times \sqrt{\left(SJC28\right)}+0.014\times GH+0.36\times \ln\left(CRP+1\right)+0.96$$

where TJC  represents  tender joint count and SJC  represents  swollen joint count. DAS-28-CRP scores were classified into four categories: inactive disease (DAS-28-CRP < 2.6), mild (DAS-28-CRP 2.6–3.2), moderate (DAS-28-CRP 3.2–5.1), and strong disease activity (DAS-28-CRP >5.1).

Evaluated comorbidities included Cushing’s syndrome, hepatitis B, hypertension, gastrointestinal disease, osteoporosis, heart disease, diabetes, and lung disease.

Latent TB screening at the time of initiation of biologic DMARDs included assessment of risk factors for latent TB infection (yes/no) and interferon gamma-release assay (IGRA) results (negative/positive). Monitoring for latent and active TB during treatment was performed based on clinical symptoms and various screening tests, as appropriate: IGRA, chest X-ray, chest computed tomography upon suspicion of TB lesion on chest X-ray, acid-fast bacteria (AFB) test, and bronchoscopy. The follow-up period in our study was defined as the duration of follow-up by the treating physician after 12 months of follow-up by the researchers. Lack of education refer to the people that having had little or no formal education at a school.

Serum rheumatoid factor (RF) levels were evaluated using a turbidimetric RF immunoassay performed at the Department of Biochemistry of Bach Mai Hospital (Vietnam). RF levels >14 IU/mL were considered to indicate positive results. Serum CRP levels were evaluated using a turbidimetric CRP immunoassay at the Department of Biochemistry of Bach Mai Hospital with Olympus AU 640 device. The IGRA test, an in vitro quantitative assay of interferon gamma, a component of the cell-mediated immune response to TB antigens, has the potential to detect TB infection even in the latent state. After collecting blood samples, 1 mL of blood was placed into each of the four test tubes (Nil, TB1, TB2, and Mitogen), which were incubated for 16–24 h. The test was performed at the Microbiology Department of Hanoi Lung Hospital. Results were interpreted using the quantiFERON software: interferon gamma levels ≥0.35 UI in TB1 or TB2 or ≥25% of the Nil result were considered to indicate positive results.

### Biologic DMARD therapy

The following biologic DMARD protocols were used for RA treatment.

1. Tocilizumab (Actemra 200mg^®^) was administered intravenously at 4 mg/kg once every 4 weeks, with dose increase up to 8 mg/kg once every 4 weeks based on the clinical response (maximum dose: 800 mg). In case of development of a serious infection, therapy was discontinued until the infection was controlled [7,10].
2. Adalimumab (Humira^®^) was administered at a dose of 40 mg every other week, with dose increase up to 40 mg every week or 80 mg every other week for select patients with an inadequate response [7].
3. Infliximab (Remicade^®^) was administered at a dose of 3 mg/kg at 0, 2, and 6 weeks as induction therapy, followed by intravenous maintenance therapy at a dose of 3 mg/kg every 8 weeks thereafter [7].
4. Golimumab (Simponi^®^) was administered at a dose of 50 mg once a month [7].

### Statistical analysis

A logistic regression model was built using R statistical software version 3.2.4 (R Core Team, Vienna, Austria). We used forward selection based on the chi-square test of the change in residual deviance. Differences among groups were analyzed using the chi-square test for qualitative variables, one-way analysis of variance for continuous variables with normal distribution, and the nonparametric Kruskal–Wallis test for continuous variables with nonstandard distribution. Analysis of variance was used to compare the differences in clinical laboratory indicators before and after biologic DMARD treatment. Comparison between baseline and after 12 weeks of treatment with DAA was performed using McNemar’s test. Differences were considered statistically significant at p ≤ 0.05.

### Ethical considerations

The study protocol was reviewed and approved by the Institutional Review Board of Bach Mai Hospital, Vietnam (Decision 1292/QĐ-BVBM). The study was conducted in accordance with the Declaration of Helsinki and International Conference on the Harmonization of the Technical Requirements for the Registration of Pharmaceuticals for Human Use—Good Clinical Practice guidelines. Prior to data collection, ethical approval was obtained from the ethics subcommittees of Bach Mai Hospital. All patients provided written informed consent prior to study commencement.

## Results

### Patient characteristics

**Fig 1:**
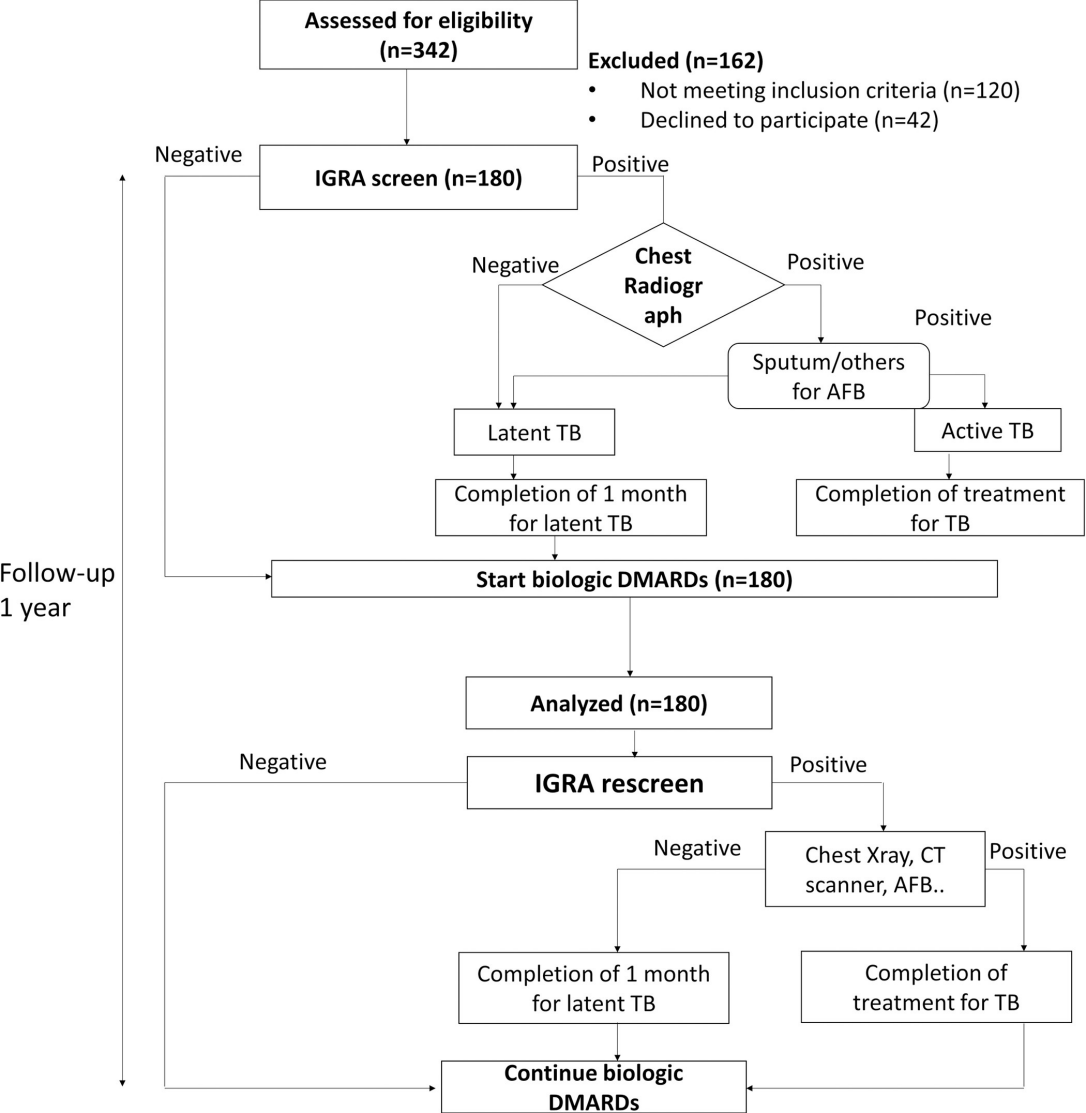
Study flowchart. TB, tuberculosis; IGRA, interferon gamma-release assay; DMARDs, disease-modifying antirheumatic drugs; AFB, acid-fast bacteria; CT, computed tomography.

Of the 342 patients screened, 162 were excluded due to not meeting the inclusion criterion or refusing to participate. Finally, 180 patients were included in the study, all of whom were followed up for at least 1 year. The data underling our study are shown in S1 File. The study flowchart is presented in Fig 1.

The mean age was 55.9 ± 10.3 years, with the highest proportion of patients in the 50–59 (35.5%) and 60–69 (35.6%) age groups, and the majority of patients were female, accounting for 87.2%. The time from RA diagnosis to initiation of biologic therapy was less than 5 years in most patients (n = 111, 61.7%). Notably, one patient started receiving biologic DMARDs at 1 month from disease diagnosis. The most common comorbidities were Cushing’s syndrome, hypertension, and osteoporosis, each accounting for more than a quarter of the study population. Notably, 4.4% of patients were diagnosed with hepatitis B. Other less common comorbidities included gastrointestinal diseases and hypothyroidism. Patients’ characteristics are described in Table 1.

**Table 1:** Patient characteristics (n = 180).

Characteristics		Results
Weight, kg, mean (SD)		51.6 (4.3)
Height, cm, mean (SD)		154.2 (5.4)
BMI, kg/m2, mean (SD)		21.7 (1.1)
Age, years, median (IQR)		55.9 (23,78)
	<40 (%)	7.8
	40–49 (%)	16.7
	50–59 (%)	35.5
	60–69 (%)	35.6
	70–79 (%)	4.4
Sex (female), n (%)		87.2
Duration of conventional DMARDs, months		31.52 (38.74)
Time of RA onset, n (%)		
< 5 years		111 (61.7)
5–10 years		26 (14.4)
≥ 10 years		43 (23.9)
Comorbidity, n (%)		
	Cushing’s syndrome	51 (28.3)
	Hypertension	44 (24.4)
	Osteoporosis	49 (27.2)
	Diabetes	29 (16.1)
	Hepatitis B	8 (4.4)
	Digestive tract disease	11 (6.1)
	Heart disease	7 (3.9)
	Lung disease	6 (3.3)

SD, standard deviation; BMI, body mass index; IQR, interquartile range; DMARDs, disease-modifying antirheumatic drugs; RA, rheumatoid arthritis.

### RA treatment before biologic therapy initiation

The most common conventional DMARD regimen before initiation of biologic therapy was methotrexate + hydroxychloroquine (n = 92, 51.1%), followed by methotrexate monotherapy (n = 57, 31.7%), and triple therapy with methotrexate + hydroxychloroquine + sulfasalazine (n = 9, 5.0%). Twelve patients were started on biologic therapy with no clear history of DMARD use (Table 2). In the majority of patients (n = 125, 69.5%), methylprednisolone was used at a dose of 4–16 mg/day (Table 2).

**Table 2:** DMARD regimen before biologic therapy initiation (n = 180).

**csDMARDs before bDMARDs, n (%)**	
Methotrexate + hydroxychloroquine	92 (51.1)
Methotrexate	57 (31.7)
Methotrexate + hydroxychloroquine + sulfasalazine	9 (5)
Methotrexate + sulfasalazine	2 (1.1)
Hydroxychloroquine + leflunomide	2 (1.1)
Hydroxychloroquine	2 (1.1)
Hydroxychloroquine + sulfasalazine	2 (1.1)
Methotrexate + leflunomide	2 (1.1)
non-DMARDs	12 (6.7)
**Use of methylprednisolone, n (%)**	
< 4 mg	16 (8.9)
4–16 mg	125 (69.5)
16–32 mg	35 (19.4)
≥ 32 mg	4 (2.2)

csDMARDs, conventional synthetic disease-modifying antirheumatic drugs; bDMARDs, biologic disease-modifying antirheumatic drugs.

### RA characteristics before biologic therapy initiation

Nearly all patients reported a moderate-intensity pain before initiating biologic therapy, with an average VAS score of 5.71. At the time of first use of biologics, most patients had a DAS-28-CRP score higher than 3.2, indicating moderate to strong disease activity. Only one patient with a DAS-28-CRP score of less than 2.6 received biologics (Table 3).

**Table 3:** RA characteristics before initiation of biologic DMARD therapy.

Criteria	Results
VAS (cm)	5.71 (0.94)
Morning stiffness (hour)	1.33 (1.1)
CRP (mg/dL)	3.75 (3.26)
Disease activity, DAS-28-CRP, mean (SD)	4.6 (0.9)
< 2.6, n (%)	1 (0.6)
2.6–3.2, n (%)	8 (4.4)
3.2–5.1, n (%)	116 (64.4)
>5.1, n (%)	55 (30.6)
RF positivity, n (%)	155 (86.1)

VAS, visual analog scale; CRP, C-reactive protein; DAS-28-CRP, disease activity score-28 based on C-reactive protein levels; SD, standard deviation; RF, rheumatoid factor; DMARD, disease-modifying antirheumatic drug.

### TB screening

All 180 patients were screened for latent TB infection prior to initiating biologic therapy. Among them, 36 (20%) patients had positive IGRA test results and all were treated for latent TB, with the most commonly prescribed regimen being isoniazid + rifampicin (n = 33, 91.6%). Tocilizumab was the most frequently selected biologic DMARD (n = 32, 88.8%). Among patients with initially negative IGRA test, 10 (6.9%) patients tested positive on IGRA retesting.

Of the total study population, 3 (1.7%) patients developed active TB during the follow-up period, two of whom had a negative initial IGRA test (1.4%) and one who had a positive initial IGRA test (2.8%). There was one case of pulmonary, pleural, and gluteal TB each (Table 4).

**Table 4:** Screening for latent and active TB infection before and during biologic therapy.

Characteristics	N (%)
Positive IGRA test before biologic DMARD therapy (n = 180)	36 (20)
Positive IGRA test during biologic DMARD therapy after negative initial IGRA (n = 144)	10 (6.9)
Active TB during biologic DMARD therapy (n = 180)	3 (1.7)
Latent TB infection regimen (n = 36)	
isoniazid + rifampicin	33 (91.6)
isoniazid (6 months)	2 (5.56)
isoniazid (9 months)	1 (2.8)
Biologic DMARDs for patients with latent TB (n = 36)	
tocilizumab	32 (88.8)
adalimumab	2 (5.6)
infliximab	1 (2.8)
golimumab	1 (2.8)

IGRA, interferon gamma-release assay; DMARDs, disease-modifying antirheumatic drugs; TB, tuberculosis.

### Factors associated with development and early diagnosis of active and latent TB infection

When considering risk factors for the occurrence of latent and active TB infection, we found a significant association for history of TB risk factor exposure and lack of education, with odds ratios (95% confidence interval [CI]) of 1.98 (1.78; 2.2) and 1.45 (1.31; 1.6), respectively (Table 5).

**Table 5:** Factors associated with development and early diagnosis of active and latent TB infection among the study population (n = 180).

Covariates	Latent tuberculosis			Tuberculosis		
	Odds ratio	95% CI	p-value	Odds ratio	95% CI	p-value
Age (years)	1.00	(1.00; 1.01)	0.119	1	(0.99; 1.00)	0.299
Weight (kg)	0.99	(0.99; 1.01)	0.992	1	(0.99; 1.00)	0.061
Height (cm)	1.00	(1; 1.01)	0.453	1	(0.99; 1.00)	0.1
BMI (kg/m2)	1.02	(0.99; 1.05)	0.210	1	(0.99; 1.02)	0.773
Follow-up duration (months)	1.00	(1; 1.01)	0.037*	0.99	(0.99; 1.00)	0.473
Number of IGRA tests (times)	1.02	(0.98; 1.06)	0.309	0.99	(0.97; 1.00)	0.2
Number of chest X-ray tests (times)	1.02	(1; 1.05)	0.048*	1	(0.99; 1.01)	0.928
Number of CT tests (times)	1.12	(1.11; 1.2)	0.001***	1.03	(1.01; 1.06)	0.007**
Number of bronchoscopy tests (times)	1.11	(1.09; 1.2)	0.001***	1.03	(1.01; 1.06)	0.007**
Number of sputum AFB test (times)	1.13	(1.09; 1.17)	0.001***	1.04	(1.02; 1.06)	0.001***
Time from RA onset to bDMARDs initiation	1.00	(1; 1)	0.213	1	(0.99; 1.00)	0.957
Tender joint count	1.00	(0.99; 1.01)	0.869	0.99	(0.99; 1.00)	0.432
Swollen joint count	1.00	(0.99; 1.01)	0.714	1	(0.99; 1.00)	0.989
VAS	0.99	(0.96; 1.03)	0.702	0.99	(0.97; 1.01)	0.485
CRP	1.00	(0.99; 1.01)	0.77	1	(0.99; 1.00)	0.549
DAS-28-CRP	1.02	(0.98; 1.06)	0.359	1	(0.98; 1.02)	0.875
Positive RF (ref: negative)	0.94	(0.85; 1.03)	0.193	0.98	(0.93; 1.04)	0.486
Exposure to TB risk factors (ref: no)	1.98	(1.78; 2.2)	0.001***	1.35	(1.26; 1.45)	0.001***
**Concomitant disease**						
Cushing’s syndrome	1.00	(0.93; 1.08)	0.904	0.98	(0.94;1.02)	0.275
Hypertension	0.96	(0.89; 1.04)	0.324	1.01	(0.97; 1.05)	0.662
Osteoporosis	0.95	(0.88; 1.03)	0.210	0.98	(0.94; 1.02)	0.288
Diabetes	1.02	(0.93; 1.11)	0.732	1.02	(0.97; 1.08)	0.416
Hepatitis B	0.94	(0.80; 1.11)	0.486	0.98	(0.89; 1.08)	0.708
Digestive tract disease	0.94	(0.82; 1.08)	0.409	0.98	(0.91; 1.06)	0.658
Heart disease	0.94	(0.79; 1.12)	0.516	0.98	(0.89; 1.08)	0.730
Lung disease	0.94	(0.78; 1.14)	0.548	0.98	(0.88; 1.09)	0.747
**Residence area**						
Grade 2 urban (ref: Grade 1 urban)	0.99	(0.92; 1.07)	0.846	1	(0.96; 1.05)	0.964
Grade 3 urban (ref: Grade 1 urban)	1.05	(0.96; 1.15)	0.319	0.98	(0.93; 1.03)	0.455
Non-educated (ref: educated)	1.45	(1.31; 1.6)	0.001***	1.18	(1.12; 1.25)	0.001***
**bDMARDs**						
adalimumab (ref: tocilizumab)	0.94	(0.83; 1.07)	0.3549	0.99	(0.93; 1.06)	0.834
infliximab (ref: tocilizumab)	0.94	(0.81; 1.09)	0.44231	1.09	(1.01; 1.19)	0.027*
golimumab (ref: tocilizumab)	1.06	(0.94; 1.19)	0.31392	1.05	(0.99; 1.12)	0.116
Positive LTB screening (ref: no)	0.93	(0.86; 1.01)	0.1049	1.01	(0.97; 1.06)	0.563
Positive IGRA after bDMARDs (ref: N1)				1.09	(1.01; 1.19)	0.034*

BMI, body mass index; IGRA, interferon gamma-release assay; CT, computed tomography; AFB, acid-fast bacteria; RA, rheumatoid arthritis; bDMARDs, biologic disease-modifying antirheumatic drugs; VAS, visual analog scale; CRP, C-reactive protein; DAS-28-CRP, disease activity score-28 based on C-reactive protein levels; RF, rheumatoid factor; TB, tuberculosis; LTB, latent tuberculosis.

Among factors that can aid in the early diagnosis of latent TB infection, a significant association was observed for follow-up duration and the number of X-ray, computed tomography, bronchoscopy, and sputum AFB examinations, with odds ratio (95% CI) of 1.00 (1; 1.01), 1.02 (1; 1.05), 1.12 (1.11; 1.2), 1.11 (1.09; 1.2), and 1.13 (1.09; 1.17), respectively. We found no association between concomitant disease including Cushing’s syndrome, hypertension, osteoporosis, diabetes, hepatitis B, digestive tract disease, heart disease, lung disease with the development of LTB as well as TB (p>0.05) (Table 5).

## Discussion

In this study, we observed a high prevalence of latent TB infection (20%) among patients with RA. Furthermore, history of TB risk factor exposure and lack of education were identified as risk factors for occurrence of active and latent TB infection, while follow-up duration and number of X-ray, computed tomography, bronchoscopy, and sputum AFB examinations were identified as factors that can aid in the early diagnosis of latent TB in this population.

The baseline characteristics of RA in our study, including the duration of conventional DMARD therapy before initiation of biologic therapy, VAS pain scores, duration of morning stiffness, and CRP levels, were similar to those reported in the AMBITION study in 2010 and the ADACTA study in 2013 [19,20]. The time from RA onset to initiation of biologic therapy in the current study ranged from 1 month to 40 years, with a mean of 70 months, and in the majority of patients, biologic therapy was initiated less than 5 years after RA diagnosis. In addition, a significant number of patients with RA for 10 years or more received biologic therapy (23.9%). Notably, there was 1 patient in whom biologic therapy was started within 1 month of RA onset. In the AMBITION study, 41% of patients prescribed tocilizumab had RA duration of less than 2 years [20]. Similarly, in the ADACTA trial, RA duration before biologic therapy initiation was 6.3 ± 6.9 years in the adalimumab arm and 7.3 ± 8.1 years in the tocilizumab arm [19]. These findings suggest low access to biologics; however, there was no delay in initiating use of biologic DMARDs for patients with RA in our study.

Among comorbidities in patients with RA treated with biologics, Cushing’s syndrome stands out as the most common (28.3%), occurring more frequently than hypertension (24.4%) and osteoporosis (27.2%). These numbers are slightly lower than those reported in the study of Kłodziński and Wisłowska, in which the prevalence of hypertension and osteoporosis was 35% and 32.6%, respectively [21]. This may be due to the lack of careful assessment and screening for comorbidities. On the other hand, the unusually high frequency of Cushing’s syndrome reflects the current alarming situation of corticosteroid abuse in patients with RA due to widespread indiscriminate use and easy access to corticosteroids without medical supervision and management. This syndrome can also worsen or even cause other conditions. Since Vietnam is in an endemic area for hepatitis B, hepatitis B was found in 8 patients (4.4%). Along with latent TB, hepatitis B is an important infectious disease that requires careful screening before starting biologics. Other less common comorbidities included gastrointestinal, pulmonary, and cardiovascular disease, and hypothyroidism, each occurring in less than 10% of patients. All comorbid conditions, despite their low prevalence, need attention for better disease management and improved quality of life.

Evaluation of disease activity in patients with RA is an important step in clinical practice, with the aim of assessing disease progression, response to treatment, and prognosis [10,12]. The mean DAS-28 was 6.8 ± 1.0 in the AMBITION and 6.8 ± 0.9 and 6.7 ± 0.9 in the ADACTA trial for the adalimumab and tocilizumab groups, respectively [19,20]. In the present study, the mean DAS-28 was lower than that in these previous studies (4.6 ± 0.9). This difference could be due to the changed timeframe. With more high-quality data on the efficacy and safety of biologics in general, as well as the more common use of these agents in the timeframe covered by this study, physicians’ confidence in prescribing these drugs is likely higher than it was a decade ago. In addition to physicians’ subjective assessment, erythrocyte sedimentation rate and CRP concentration are two important, widely used tests to help provide more objective parameters to physicians. Various combinations of these measures have been developed to form several composite indices for the assessment of clinical disease activity, of which the most commonly used include the DAS, simple disease activity index, and clinical disease activity index. The patients in this study, similarly to those in previous studies on biologic drugs, all had a steady increase in the index as they approached the late stage of the disease. At the time biologics were introduced, the majority of patients had a DAS-28 higher than 3.2, corresponding to moderate to high disease activity. Only one patient had a DAS-28 lower than 2.6, while 30.6% of patients had high disease activity (DAS-28 > 5.1). A high DAS-28 index reflects a high level of disease activity and indicates that the patient has a high disease burden and high risk of disability. Scott et al. reported persistent physical disability and poor quality of life in patients with RA with persistent moderate disease activity [22].

RF is a poor prognostic factor in RA. RF-positive patients with RA may have more severe early erosive arthritis and extra-articular manifestations than RF-negative patients [23,24]. In the current study, the proportion of RF-positive patients was 86%, which is higher than the RF-positivity rate reported in previous studies. In the ADACTA trial, this rate was 73% in the adalimumab and 75% in the tocilizumab arm [19]. The quantitative level of RF should be considered when analyzing its utility. The higher the quantitative result, the greater the probability that the patient has RA. In combination with other indices, such as the anti-cyclic citrullinated peptide, CRP, and erythrocyte sedimentation rate, and severity of synovitis on physical examination, RF testing can be useful to predict progression of synovitis and radiological changes and to guide treatment in patients with RA. The RF status may be of value in predicting the response to treatment and comorbidities. For example, the monoclonal antibody against the CD20 antigen on the surface of B lymphocytes, rituximab, may be less effective in seronegative than in seropositive patients with RA [25]. In contrast, anti-TNF therapy may be less effective in seropositive than in seronegative RA [26]. In addition, RF positivity in patients with RA may increase the risk of cardiovascular disease [25]. In this study, we did not find a statistically significant association between RF positivity and the occurrence of active and latent TB infection.

Most patients in our study had a clear history of using classical DMARDs for the treatment of RA before starting biologics, and the most common regimen was methotrexate + hydroxychloroquine. However, 12 patients treated with biologics had no clear history of DMARD use. Methotrexate was used in most patients. This confirms the importance of this drug already reported in previous studies. Randomized head-to-head trials have found that methotrexate monotherapy has an earlier onset of action, equal or greater efficacy, better long-term effect, and, as a result, improved survival compared with other conventional non-biologic DMARD monotherapies [27,28]. Direct comparison of methotrexate with TNF inhibitor monotherapy has also shown similar clinical benefits, with ACR criteria 20, 50, and 70 after 6–12 months of approximately 60, 40, and 20% [29,30].

In the present study, the daily systemic corticosteroid dose was maintained in 92.3% of patients, which is a 47–57% higher proportion than that reported in the AMBITION and ADACTA trials in all study groups [19,20]. While methotrexate is an important DMARD, corticosteroids remain important in the initial treatment of patients with highly active RA. The efficacy of short-term, low-dose glucocorticoids in this case has been demonstrated in randomized trials and in a large number of observational studies [31,32]. However, the use of glucocorticoids is associated with a risk of several diseases, including osteoporosis, diabetes mellitus, and adrenal insufficiency; therefore, monitoring and dose adjustment is required for patients who will be receiving these drugs. In addition, as RA is also considered an independent risk factor for osteoporotic fracture, fracture risk assessment should be performed to help make treatment decisions.

From 2017 to 2022, four biologic drugs were used to start biologic therapy at our hospital, of which tocilizumab accounted for the highest proportion (76.7%). Due to its relatively lower cost, reasonable time distance between doses, and good safety and efficacy, tocilizumab has become the biologic of choice for three-quarters of patients with RA. Tocilizumab is a monoclonal antibody directed against membrane and soluble forms of the IL-6 receptor. IL-6 is a proinflammatory cytokine involved in the pathogenesis of RA that affects both inflammation and joint damage. Its receptor binding activates intracellular signaling pathways that influence the acute phase response, cytokine production, and osteoclast activation. Clinical trials have demonstrated the clinical efficacy of tocilizumab therapy for RA, both as monotherapy and in combination with methotrexate and other DMARDs. In ADACTA, a randomized, double-blind, phase 4 controlled trial comparing tocilizumab and adalimumab monotherapy, the mean 24-week change from baseline in DAS-28 in the tocilizumab arm (-3.3) was significantly greater than that in the adalimumab arm (-1.8) (difference -1.5, 95% CI -1.8 to -1.1; p<0.0001) [19].

In our study, 20% of the patients had positive IGRA test results prior to initiating biologics, which is a much higher proportion than the 10% reported in the study of Nisar et al. in the United Kingdom [33]. This can be explained by the fact that the United Kingdom is a developed country in terms of both economy, society, and health, greatly surpassing the common level of the world in general and Vietnam in particular. However, the prevalence of latent TB in the community published by the Asian Council of Latent Tuberculosis Experts held in 2018 in Singapore is much higher. This panel of 13 TB experts from Bangladesh, Cambodia, Hong Kong, India, Indonesia, Malaysia, Myanmar, Philippines, Singapore, Taiwan, Thailand, and Vietnam reported potential TB prevalence rates. The highest latent TB rates of approximately 31% and 28% were reported in Southeast Asia and the Western Pacific region, respectively [34]. This shows that screening for latent TB before biologic therapy is extremely important, particularly in developing countries with high TB prevalence like Vietnam. Although the prevalence of latent TB in our study is similar to that previously reported in Asian countries, our study is distinct in that it provides information on the prevalence of latent TB before the start of DMARD therapy (20%), as well as the incidence of latent TB during DMARD therapy (6.9%) in patients with RA. This information will be helpful to clinicians in the monitoring and treatment of patients with RA. In addition, studies evaluating risk factors for active and latent TB have only analyzed factors such as DMARDs and comorbid conditions, while the role of TB exposure, educational status, residence, follow-up duration, and number of paraclinical tests has not been assessed. Although the ACR recommendation still mentions the Mantoux test for latent TB screening, in our study, no patients were screened by this test. The two currently available methods for diagnosis of latent TB include the century-old Mantoux tuberculin skin test (TST) and decade-old immunodiagnostic test, interferon gamma-release assay (IGRA). Both these tests work on the principle of cell-mediated immunity. In an attempt to overcome the limitations of these tests, IGRAs utilizing region of difference-1 (RD-1) *Mycobacterium tuberculosis-*specific antigen were developed, which are claimed to be more specific than TST. However, due to the absence of gold standard, there are limited data on the diagnostic performance of these tests in latent TB. Studies comparing the performance of these tests either using surrogate measures of sensitivity or specificity or index of exposure as the reference standard are not clinically relevant; thus, it remains unclear which test better identifies latent TB [35,36].

In the present study, all 36 patients with a positive IGRA test received anti-TB therapy for 1 month before starting biologics for the first time, which is fully consistent with the 2012 and 2021 ACR guidelines [7,37]. The most commonly used regimen was isoniazid + rifampicin. According to the recommendations of the Centers for Disease Control and Prevention, the regimen of rifampicin combined with isoniazid is not inferior to isoniazid treatment for 6–9 months. Isoniazid + rifampicin combination regimen dominates almost completely due to the short treatment time (3 months), thereby increasing the completion rate of patients and reducing the risk of liver damage as well as the risk of drug discontinuation due to other adverse events [38]. In a clinical trial conducted in the USA, Canada, Brazil, and Spain, Sterling et al. concluded that the use of rifampicin in combination with isoniazid for 3 months was as effective as 9 months of isoniazid alone in preventing TB and had a higher treatment completion rate [39].

We identified 3 patients (1.7%) who developed active TB during the follow-up period, two from the IGRA-negative group and one from the IGRA-positive group. This result is similar to that of Slouma et al., who reported that among 113 patients, two patients from the TB-negative group and none of the patients from the latent TB group developed active TB after 3.91 years of follow-up [40]. Similarly, in the study of Hanta et al., which included 192 patients with latent TB who received biologics between April 2005 and August 2008, only 3 (1.6%) patients developed active TB during the study period, all of whom were in the non-TB treatment group [41]. In all three cases in the current study, the definitive diagnosis of active TB was based on AFB results. The AFB test was shown to be extremely useful, particularly for abscesses suspected of extrapulmonary TB. These are often considered by clinicians as bacterial skin infections (mostly caused by *Staphylococcus aureus*), and diagnostic focus is placed only on culture tests, ignoring the AFB test. Based on these results, TB treatment for latent TB-positive patients during screening before initiation of biologic DMARD therapy is highly effective in active TB prevention.

We identified history of TB risk factor exposure and lack of education as risk factors for occurrence of active and latent TB infection. According to the 2022 World Health Organization report, Vietnam ranks 10^th^ among 30 countries with a high TB burden and 11^th^ among 30 countries with the highest burden of multidrug-resistant TB [42]. In our study, only 10 patients (5.6%) had exposure to risk factors for latent TB infection, and for all of them, the risk factor was household exposure to someone with TB. Given the current TB situation in Vietnam, this number may not reflect all patients in contact with patients with pulmonary TB. Despite persistent cough symptoms, the diagnosis of pulmonary TB remains difficult, particularly in rural areas, due to the limited knowledge of a part of the population who still believe that TB is a genetic disease. Indeed, our findings indicated that patients’ educational level is an important predictor of TB infection. The reason for this may be that patients with lack of education often delay initiation of treatment despite receiving a diagnosis or appropriate guidance during the first consultation. Possible reasons reported are seeking a second or even third opinion due to the psychological factor of denial among patients diagnosed with this stigmatizing disease [43,44]. Our study found no difference between biological DMARDs for development of LTB and active TB. Agree with us, in a cohort study that included 951 patients with RA in Taiwan, the authors found no significant difference in the risk of TB between adalimumab and etanercept users; however, they found that treatment with sulfasalazine appeared to have a protective effect (HR: 0.32, 95% CI: 0.11–0.97, p = 0.043) [45]. Our study had several limitations. First, we could not accurately assess patients’ adherence to TB medication because the majority of patients were treated on an outpatient basis. However, patients were asked about using the prescription drugs at each follow-up visit. Second, other risk factors affecting the development of TB were not evaluated, such as nutritional status, substance abuse, and smoking tobacco. Finally, the variability in the process of ordering X-ray and CT based on the indications likely introduced selection bias. Patients with positive IGRA test results undergo X-ray and CT examinations, and these tests are repeated several times during RA treatment. Variability is introduced due to differences in the procedure selection process based on characteristics like comorbidities or prognostic factors [46]. However, in the multivariable analysis for factors predicting the occurrence of TB and latent TB, the X-ray and CT variables were quantitative, not qualitative variables (yes/no), clearly showing a relationship between the number of tests performed and early diagnosis of TB and latent TB, thus confirming the reliability of the established TB and latent TB diagnosis and prognosis procedures.

## Conclusions

Our study found a high prevalence of latent TB infection among patients using biologic DMARDs of 20%, while only 1.7% of patients developed active TB after a 12-month follow-up. History of TB risk factor exposure and lack of education were significantly associated with the occurrence of active and latent TB infection, whereas follow-up duration and number of X-ray, computed tomography, bronchoscopy, and sputum AFB examinations were identified as factors that can aid in the early diagnosis of latent TB. These findings indicate that latent TB infection has a high prevalence among patients with RA in countries with high TB burden like Vietnam. We also provide useful information for the screening, monitoring, and treatment of latent and active TB infection in patients with RA.

## Supporting information

S1 File Study data. (XLSX)

## References

[1] Gabriel SE. The epidemiology of rheumatoid arthritis. Rheum Dis Clin North Am. 2001;27(2):269–81. doi: 10.1016/s0889-857x(05)70201-5 11396092

[2] Silman AJ, Pearson JE. Epidemiology and genetics of rheumatoid arthritis. Arthritis Res. 2002;4 Suppl 3(Suppl 3):S265–72. doi: 10.1186/ar578 12110146 PMC3240153

[3] Alamanos Y, Voulgari PV, Drosos AA. Incidence and prevalence of rheumatoid arthritis, based on the 1987 American College of Rheumatology criteria: a systematic review. Semin Arthritis Rheum. 2006;36(3):182–8. doi: 10.1016/j.semarthrit.2006.08.006 17045630

[4] Arnett FC, Edworthy SM, Bloch DA, McShane DJ, Fries JF, Cooper NS, et al. The American Rheumatism Association 1987 revised criteria for the classification of rheumatoid arthritis. Arthritis Rheum. 1988;31(3):315–24. doi: 10.1002/art.1780310302 3358796

[5] Silman AJ. The 1987 revised American Rheumatism Association criteria for rheumatoid arthritis. Br J Rheumatol. 1988;27(5):341–3. doi: 10.1093/rheumatology/27.5.341 3179623

[6] Aletaha D, Neogi T, Silman AJ, Funovits J, Felson DT, Bingham CO, et al. 2010 Rheumatoid arthritis classification criteria: an American College of Rheumatology/European League Against Rheumatism collaborative initiative. Arthritis Rheum. 2010;62(9):2569–81. doi: 10.1002/art.27584 20872595

[7] Fraenkel L, Bathon JM, England BR, St Clair EW, Arayssi T, Carandang K, et al. 2021 American College of Rheumatology Guideline for the Treatment of Rheumatoid Arthritis. Arthritis Rheumatol. 2021;73(7):1108–23. doi: 10.1002/art.41752 34101376

[8] Benucci M, Saviola G, Manfredi M, Sarzi-Puttini P, Atzeni F. Cost effectiveness analysis of disease-modifying antirheumatic drugs in rheumatoid arthritis. A systematic review literature. Int J Rheumatol. 2011;2011:845496. doi: 10.1155/2011/845496 22162693 PMC3228304

[9] Joensuu JT, Huoponen S, Aaltonen KJ, Konttinen YT, Nordström D, Blom M. The cost-effectiveness of biologics for the treatment of rheumatoid arthritis: a systematic review. PLoS One. 2015;10(3):e0119683. doi: 10.1371/journal.pone.0119683 25781999 PMC4363598

[10] Smolen JS, Landewé R, Bijlsma J, Burmester G, Chatzidionysiou K, Dougados M, et al. EULAR recommendations for the management of rheumatoid arthritis with synthetic and biological disease-modifying antirheumatic drugs: 2016 update. Ann Rheum Dis. 2017;76(6):960–77. doi: 10.1136/annrheumdis-2016-210715 28264816

[11] Hazlewood GS, Barnabe C, Tomlinson G, Marshall D, Devoe DJ, Bombardier C. Methotrexate monotherapy and methotrexate combination therapy with traditional and biologic disease modifying anti-rheumatic drugs for rheumatoid arthritis: A network meta-analysis. Cochrane Database Syst Rev. 2016;2016(8):CD010227. 10.1002/14651858.CD010227.pub2 PMC7087436 27571502

[12] Smolen JS, Emery P, Fleischmann R, van Vollenhoven RF, Pavelka K, Durez P, et al. Adjustment of therapy in rheumatoid arthritis on the basis of achievement of stable low disease activity with adalimumab plus methotrexate or methotrexate alone: the randomised controlled OPTIMA trial. Lancet. 2014;383(9914):321–32. doi: 10.1016/S0140-6736(13)61751-1 24168956

[13] Nisar MK, Rafiq A, Östör AJ. Biologic therapy for inflammatory arthritis and latent tuberculosis: real world experience from a high prevalence area in the United Kingdom. Clin Rheumatol. 2015;34(12):2141–5. doi: 10.1007/s10067-015-3099-3 26497501

[14] Cantini F, Nannini C, Niccoli L, Iannone F, Delogu G, Garlaschi G, et al. Guidance for the management of patients with latent tuberculosis infection requiring biologic therapy in rheumatology and dermatology clinical practice. Autoimmun Rev. 2015;14(6):503–9. doi: 10.1016/j.autrev.2015.01.011 25617816

[15] Organization GWH. Latent tuberculosis infection: updated and consolidated guidelines for programmatic management. 2018. 30277688

[16] Haefeli M, Elfering A. Pain assessment. Eur Spine J. 2006;15 Suppl 1(Suppl 1):S17–24. doi: 10.1007/s00586-005-1044-x 16320034 PMC3454549

[17] Fransen J, van Riel PL. The Disease Activity Score and the EULAR response criteria. Rheum Dis Clin North Am. 2009;35(4):745–57, vii-viii. doi: 10.1016/j.rdc.2009.10.001 19962619

[18] Wells G, Becker JC, Teng J, Dougados M, Schiff M, Smolen J, et al. Validation of the 28-joint Disease Activity Score (DAS28) and European League Against Rheumatism response criteria based on C-reactive protein against disease progression in patients with rheumatoid arthritis, and comparison with the DAS28 based on erythrocyte sedimentation rate. Ann Rheum Dis. 2009;68(6):954–60. doi: 10.1136/ard.2007.084459 18490431 PMC2674547

[19] Gabay C, Emery P, van Vollenhoven R, Dikranian A, Alten R, Pavelka K, et al. Tocilizumab monotherapy versus adalimumab monotherapy for treatment of rheumatoid arthritis (ADACTA): a randomised, double-blind, controlled phase 4 trial. Lancet. 2013;381(9877):1541–50. doi: 10.1016/S0140-6736(13)60250-0 23515142

[20] Jones G, Sebba A, Gu J, Lowenstein MB, Calvo A, Gomez-Reino JJ, et al. Comparison of tocilizumab monotherapy versus methotrexate monotherapy in patients with moderate to severe rheumatoid arthritis: the AMBITION study. Ann Rheum Dis. 2010;69(1):88–96. doi: 10.1136/ard.2008.105197 19297346 PMC3747519

[21] Klodzinski L, Wislowska M. Comorbidities in rheumatic arthritis. Reumatologia. 2018;56(4):228–33. doi: 10.5114/reum.2018.77974 30237627 PMC6142024

[22] Scott IC, Mount J, Barry J, Kirkham B. Factors associated with disability in patients with rheumatoid arthritis with persistent moderate disease activity: a retrospective cohort study. BMC Rheumatol. 2020;4:63. doi: 10.1186/s41927-020-00161-4 33094270 PMC7576705

[23] Carpenter L, Nikiphorou E, Sharpe R, Norton S, Rennie K, Bunn F, et al. Have radiographic progression rates in early rheumatoid arthritis changed? A systematic review and meta-analysis of long-term cohorts. Rheumatology (Oxford). 2016;55(6):1053–65. doi: 10.1093/rheumatology/kew004 26961746

[24] Aletaha D, Alasti F, Smolen JS. Rheumatoid factor determines structural progression of rheumatoid arthritis dependent and independent of disease activity. Ann Rheum Dis. 2013;72(6):875–80. doi: 10.1136/annrheumdis-2012-201517 22798565

[25] Edwards JC, Cambridge G. Prospects for B-cell-targeted therapy in autoimmune disease. Rheumatology (Oxford). 2005;44(2):151–6. doi: 10.1093/rheumatology/keh446 15509628

[26] Takeuchi T, Miyasaka N, Inui T, Yano T, Yoshinari T, Abe T, et al. High titers of both rheumatoid factor and anti-CCP antibodies at baseline in patients with rheumatoid arthritis are associated with increased circulating baseline TNF level, low drug levels, and reduced clinical responses: a post hoc analysis of the RISING study. Arthritis Res Ther. 2017;19(1):194. doi: 10.1186/s13075-017-1401-2 28865493 PMC5581496

[27] Maetzel A, Wong A, Strand V, Tugwell P, Wells G, Bombardier C. Meta-analysis of treatment termination rates among rheumatoid arthritis patients receiving disease-modifying anti-rheumatic drugs. Rheumatology (Oxford). 2000;39(9):975–81. doi: 10.1093/rheumatology/39.9.975 10986302

[28] Choi HK, Hernan MA, Seeger JD, Robins JM, Wolfe F. Methotrexate and mortality in patients with rheumatoid arthritis: a prospective study. Lancet. 2002;359(9313):1173–7. doi: 10.1016/S0140-6736(02)08213-2 11955534

[29] Breedveld FC, Weisman MH, Kavanaugh AF, Cohen SB, Pavelka K, van Vollenhoven R, et al. The PREMIER study: A multicenter, randomized, double-blind clinical trial of combination therapy with adalimumab plus methotrexate versus methotrexate alone or adalimumab alone in patients with early, aggressive rheumatoid arthritis who had not had previous methotrexate treatment. Arthritis Rheum. 2006;54(1):26–37. doi: 10.1002/art.21519 16385520

[30] Bathon JM, Martin RW, Fleischmann RM, Tesser JR, Schiff MH, Keystone EC, et al. A comparison of etanercept and methotrexate in patients with early rheumatoid arthritis. N Engl J Med. 2000;343(22):1586–93. doi: 10.1056/NEJM200011303432201 11096165

[31] Conn DL. Resolved: Low-dose prednisone is indicated as a standard treatment in patients with rheumatoid arthritis. Arthritis Rheum. 2001;45(5):462–7. doi: 10.1002/1529-0131(200110)45:5&lt;462::aid-art366&gt;3.0.co;2-v 11642646

[32] Gotzsche PC, Johansen HK. Meta-analysis of short-term low dose prednisolone versus placebo and non-steroidal anti-inflammatory drugs in rheumatoid arthritis. BMJ. 1998;316(7134):811–8. doi: 10.1136/bmj.316.7134.811 9549450 PMC28482

[33] Nisar MK, Rafiq A, Ostor AJ. Biologic therapy for inflammatory arthritis and latent tuberculosis: real world experience from a high prevalence area in the United Kingdom. Clin Rheumatol. 2015;34(12):2141–5. doi: 10.1007/s10067-015-3099-3 26497501

[34] Paton NI, Borand L, Benedicto J, Kyi MM, Mahmud AM, Norazmi MN, et al. Diagnosis and management of latent tuberculosis infection in Asia: Review of current status and challenges. Int J Infect Dis. 2019;87:21–9. doi: 10.1016/j.ijid.2019.07.004 31301458

[35] Adetifa IM, Lugos MD, Hammond A, Jeffries D, Donkor S, Adegbola RA, et al. Comparison of two interferon gamma release assays in the diagnosis of Mycobacterium tuberculosis infection and disease in The Gambia. BMC Infect Dis. 2007;7:122. 17961228 10.1186/1471-2334-7-122 PMC2216027

[36] Hill PC, Fox A, Jeffries DJ, Jackson-Sillah D, Lugos MD, Owiafe PK, et al. Quantitative T cell assay reflects infectious load of Mycobacterium tuberculosis in an endemic case contact model. Clin Infect Dis. 2005;40(2):273–8. doi: 10.1086/427030 15655747

[37] Singh JA, Furst DE, Bharat A, Curtis JR, Kavanaugh AF, Kremer JM, et al. 2012 update of the 2008 American College of Rheumatology recommendations for the use of disease-modifying antirheumatic drugs and biologic agents in the treatment of rheumatoid arthritis. Arthritis Care Res (Hoboken). 2012;64(5):625–39. doi: 10.1002/acr.21641 22473917 PMC4081542

[38] Sterling TR, Njie G, Zenner D, Cohn DL, Reves R, Ahmed A, et al. Guidelines for the Treatment of Latent Tuberculosis Infection: Recommendations from the National Tuberculosis Controllers Association and CDC, 2020. MMWR Recomm Rep. 2020;69(1):1–11. doi: 10.15585/mmwr.rr6901a1 32053584 PMC7041302

[39] Sterling TR, Villarino ME, Borisov AS, Shang N, Gordin F, Bliven-Sizemore E, et al. Three months of rifapentine and isoniazid for latent tuberculosis infection. N Engl J Med. 2011;365(23):2155–66. doi: 10.1056/NEJMoa1104875 22150035

[40] Slouma M, Mahmoud I, Saidane O, Bouden S, Abdelmoula L. Latent tuberculosis infection screening prior to biological treatment in Tunisian patients. Therapie. 2017;72(5):573–8. doi: 10.1016/j.therap.2017.02.002 28318613

[41] Hanta I, Ozbek S, Kuleci S, Kocabas A. The evaluation of latent tuberculosis in rheumatologic diseases for anti-TNF therapy: experience with 192 patients. Clin Rheumatol. 2008;27(9):1083–6. doi: 10.1007/s10067-008-0867-3 18320137

[42] Bagcchi S. WHO’s Global Tuberculosis Report 2022. Lancet Microbe. 2023;4(1):e20. doi: 10.1016/S2666-5247(22)00359-7 36521512

[43] Ehsanul Huq K, Moriyama M, Zaman K, Chisti MJ, Long J, Islam A, et al. Health seeking behaviour and delayed management of tuberculosis patients in rural Bangladesh. BMC Infect Dis. 2018;18(1):515. doi: 10.1186/s12879-018-3430-0 30314453 PMC6186095

[44] Nogueira BMF, Rolla VC, Akrami KM, Kiene SM. Factors associated with tuberculosis treatment delay in patients co-infected with HIV in a high prevalence area in Brazil. PLoS One. 2018;13(4):e0195409. doi: 10.1371/journal.pone.0195409 29624603 PMC5889181

[45] Lim CH, Chen HH, Chen YH, Chen DY, Huang WN, Tsai JJ, et al. The risk of tuberculosis disease in rheumatoid arthritis patients on biologics and targeted therapy: A 15-year real world experience in Taiwan. PLoS One. 2017;12(6):e0178035. doi: 10.1371/journal.pone.0178035 28570568 PMC5453436

[46] Sackett DL. Bias in analytic research. J Chronic Dis. 1979;32(1–2):51–63. doi: 10.1016/0021-9681(79)90012-2 447779

